# A critical reappraisal of the carotid sinus and carotid bulb: Distinguishing neurohistological function from vascular geometry

**DOI:** 10.1002/ar.70149

**Published:** 2026-02-03

**Authors:** Răzvan Costin Tudose, Nicolae Radu, Mugurel Constantin Rusu

**Affiliations:** ^1^ Division of Anatomy, Department 1, Faculty of Dentistry “Carol Davila” University of Medicine and Pharmacy Bucharest Romania

**Keywords:** atherosclerosis, baroreceptors, carotid bulb, carotid sinus, hemodynamics

## Abstract

For over a century, the terms “carotid bulb” and “carotid sinus” have been used inconsistently across anatomy, imaging, and clinical practice, resulting in terminological ambiguity with implications for diagnostic reporting and procedural communication. This review establishes that these are distinct, non‐equivalent entities, and that their conflation undermines diagnostic precision and procedural safety. The carotid bulb (CB) is a variable geometric dilation of the carotid bifurcation, defined by luminal expansion and hemodynamic significance. Its fusiform widening creates regions of low, oscillatory wall shear stress that predispose to atherosclerotic plaque formation along the posterolateral internal carotid artery. In contrast, the carotid sinus (CS) is a neurohistological construct, a specialized baroreceptor field concentrated predominantly on the medial wall of the proximal internal carotid artery. These mechanosensitive nerve terminals transduce circumferential wall strain into arterial pressure signals, forming the afferent limb of the baroreflex arc. Critically, the CB and CS exhibit spatial dissociation: baroreceptors are concentrated medially, where hemodynamic forces are benign, whereas atherosclerotic disease develops posterolaterally, where flow disturbance is maximal. Immunohistochemical mapping confirms that baroreceptor density does not correlate with CB geometry. This mismatch explains variable hemodynamic responses during carotid interventions and the failure of imaging‐based anatomical landmarks to predict baroreflex function. The clinical consequences are tangible: conflated terminology obscures dual pathology in risk stratification, complicates surgical planning during carotid endarterectomy and stenting, and undermines precision in baroreflex activation therapy. We advocate for systematic adoption of function‐based terminology: “carotid bulb” for geometric features, and “carotid sinus” strictly for the neural receptor zone.

## Graphical abstract

This review redefines the carotid bulb (CB) as a variable geometric dilation shaped by hemodynamics and the carotid sinus (CS) as a conserved neurohistological baroreceptor field. Distinguishing these entities clarifies a century of anatomical confusion and links geometry, neurohistology, and clinical interpretation within a unified framework.

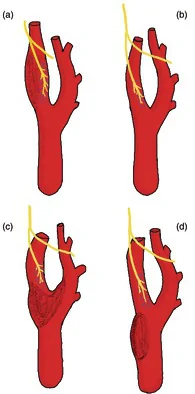

## 1 INTRODUCTION: A CRITICAL DISTINCTION IN CARDIOVASCULAR ANATOMY AND PHYSIOLOGY

Precision in terminology is central to clinical safety and scientific clarity, particularly at the carotid bifurcation, a region of immense physiological importance and pathological vulnerability. For more than a century, the terms “carotid bulb” (CB) and “carotid sinus” (CS) have been used interchangeably, and often synonymously, in medical literature, clinical practice, and educational settings (Andani & Khan, 2025; Baz et al., 2022; Gianaros et al., 2002; Idbohrn, 1951; Paturet, 1951; Seong et al., 2015; Standring et al., 2020; Webster et al., 1956). This widespread conflation reflects a longstanding conceptual ambiguity with essential consequences for anatomical reporting and clinical interpretation (see Box 1).

**BOX 1 Key terminology and recommended usage**

**Carotid bulb (CB)**. A macroscopic geometric dilatation at the carotid bifurcation, typically involving the distal common carotid artery and/or proximal internal carotid artery (variable in extent, and may be subtle or absent). It is identifiable on routine imaging (ultrasound/CTA/MRA). It shapes local flow patterns (e.g., low/oscillatory wall shear stress) and contributes to plaque predilection and procedural/hemodynamic planning.

**Carotid sinus (CS)**. A functionally defined baroreceptor field composed of mechanosensitive sensory endings in the arterial wall, concentrated predominantly along the medial aspect of the proximal internal carotid artery, with variable extension to adjacent segments. It is defined by neurohistology and afferent innervation rather than by consistent gross morphology, and it is not reliably delineated on routine imaging.

**Recommended reporting language**. Use **CB** for geometric configuration, luminal expansion, imaging‐based descriptions, and hemodynamic/atherosclerosis‐related contexts. Reserve **CS** (or “carotid baroreceptor field”) for baroreceptor distribution, baroreflex physiology, CS syndrome, and neuromodulatory targeting.

The principal morphological variants of the CB and their relationship to the CS baroreceptor field are illustrated in Figure 1.

**FIGURE 1:**
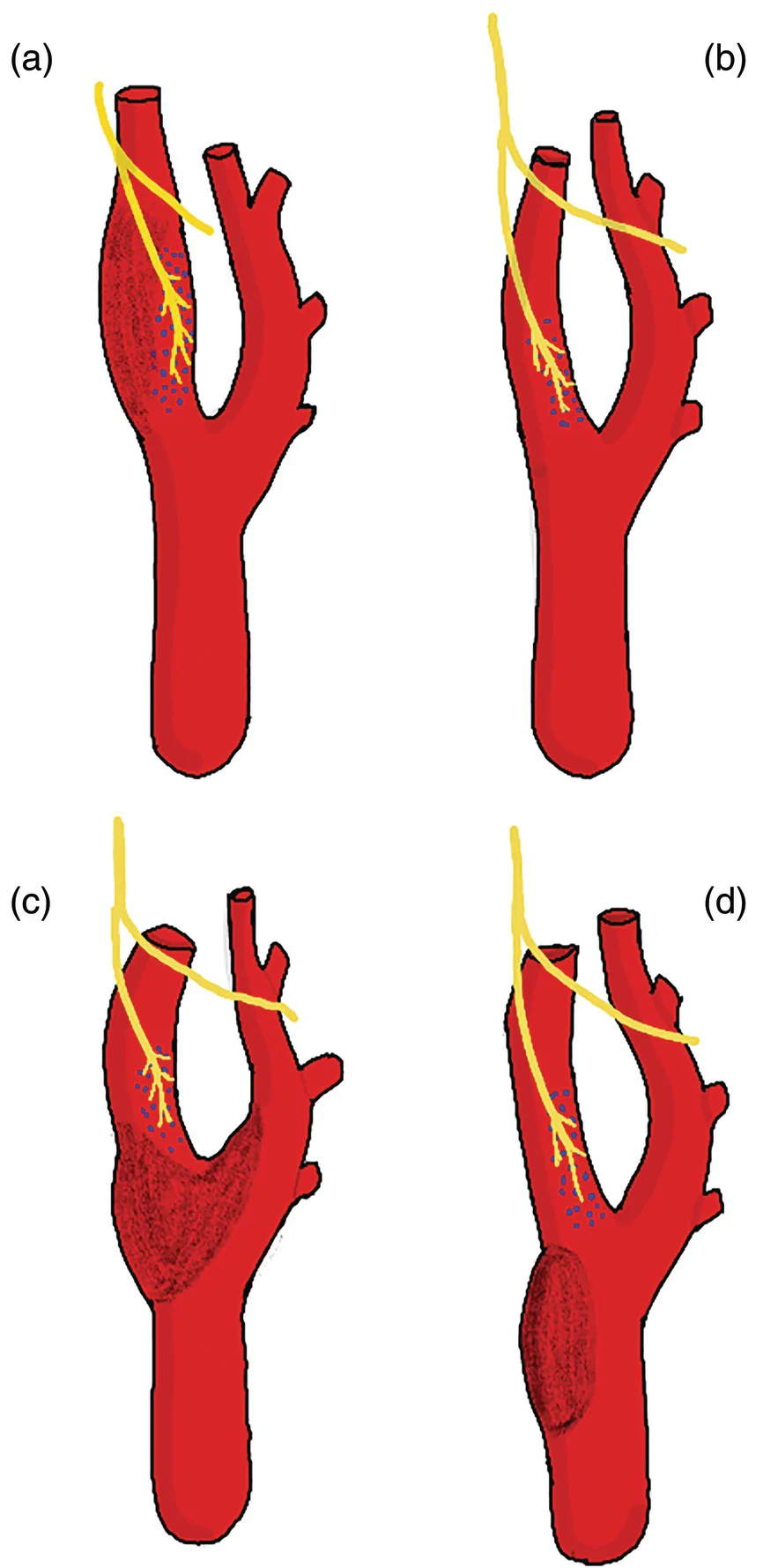
Common morphological variants of the carotid bulb (CB) and spatial relation to the carotid sinus baroreceptor field. Original schematic series (a–d) showing the four main configurations of the CB at the bifurcation on the right side. Blue dots mark carotid sinus (CS) baroreceptors within the adventitia, concentrated along the medial wall of the proximal ICA. The yellow branch represents the glossopharyngeal nerve (IX) and its carotid sinus nerve (Hering's) afferents. Dashed red stippling denotes disturbed, low‐ and oscillatory‐shear flow zones. (a) ICA bulb: focal dilation of the proximal ICA immediately distal to the flow divider. (b) Absent bulb: no discernible focal dilation. (c) Trifurcation bulb: expansion spanning the distal CCA and the origins of both ICA and ECA; the disturbed‐flow region is broad, but the baroreceptor field remains medial. (d) CCA (pre‐bifurcation) bulb: dilation confined to the distal CCA.

## 2 THE PROBLEM OF CONFLATION

Conflation of the CB and CS is widespread in classic and modern reference works, where visible bifurcation dilatation is often labeled “carotid sinus” and implicitly equated with baroreceptor location (detailed in Box 1). This synonymy was historically pragmatic, as the macroscopic geometry is readily identifiable, whereas the microscopic baroreceptive field is inaccessible to gross examination; however, this conflation is suboptimal for contemporary practice. Representative examples are summarized in Table 1.

**TABLE 1:** Examples of carotid bulb–carotid sinus conflation in commonly cited anatomical and imaging reference texts.

Author	Key description	Interpretation of usage
Paturet (Paturet, 1951)	“le sinus ou bulbe carotidien” (the sinus or CB)	Treats the terms “sinus” and “bulb” as interchangeable synonyms
Chevrel (Chevrel & Fontaine, 1996)	“…c'est. le sinus carotidien, où sont situés des baro‐récepteurs…”	Equates the slight terminal dilatation of the CCA with the CS and explicitly locates baroreceptors there
Gray's Anatomy (Standring et al., 2020)	“This dilation is the carotid sinus”	Defines the CS by geometric appearance (dilation at the bifurcation), implicitly equating visible morphology with CS location
Last's anatomy (Sinnatamby & Last, 2011)	“a slight dilatation, the carotid sinus”	Identifies the carotid sinus as a slight dilatation, reinforcing a geometry‐based definition commonly associated with baroreceptor function
Bergman's encyclopedia (Tubbs et al., 2016)	Notes the ICA origin “usually has a carotid sinus”	Uses “carotid bulb” and “carotid sinus” in a way that implies synonymy at the ICA origin
Osborn (Osborn, 1999)	Localizes CB as a “focal dilatation at the ICA takeoff”	Defines the CB by angiographic topography and contour without reference to baroreceptor content (no explicit CB–CS overlap implied)

*Note*: The table highlights definitions of gross arterial dilatation that implicitly or explicitly link geometry to baroreceptor location, thereby illustrating a persistent and anatomically inaccurate terminological practice.

Abbreviations: CB, carotid bulb; CCA, common carotid artery; CS, carotid sinus; ICA, internal carotid artery.

A key qualification appears in Hollinshead's *Anatomy for Surgeons*, which notes that the *gross* “sinus” (often termed the bulb) may not correspond to the *physiological* sinus because nerve endings can extend beyond the visible dilatation, highlighting the conceptual weakness of defining the CS by geometry alone (Hollinshead, 1982). The problem is compounded by *Terminologia Anatomica* (2019), which omits “carotid bulb” but lists “carotid sinus” as a structure of the common carotid artery (CCA) (FIPAT, 2019). This designation does not reflect the predominant neurohistological localization of baroreceptors at the proximal internal carotid artery (ICA). For educational and reporting purposes, this discrepancy can be resolved by restricting “carotid sinus” to the histologically defined baroreceptor field, while using “carotid bulb” as an imaging‐ and geometry‐based descriptor of bifurcation morphology.

## 3 DEVELOPMENTAL AND ONTOGENETIC DISSOCIATION OF THE CAROTID BULB AND CAROTID SINUS

The carotid bifurcation occupies a common embryologic vascular domain, yet CB and CS arise through different developmental programs, explaining why CB geometry does not predict baroreceptor distribution. The CCA and proximal ICA, the segments in which bulbous geometry may occur, derive primarily from the third pharyngeal (aortic) arch, while the distal ICA arises from the dorsal aorta during weeks 4–5 of pharyngeal arch remodeling (Kameda, 2020; Moore et al., 2011; Sadler, 2018). Embryonic arteries are relatively uniform tubes, and the focal dilatation that defines the CB is absent in fetal and early postnatal anatomy (Seong et al., 2005; Seong et al., 2015).

### 3.1 Postnatal emergence and mechanoadaptive remodeling of the carotid bulb

At birth, the bifurcation is a branch point without focal luminal expansion; throughout infancy and childhood, the ICA enlarges with brain growth and rising cerebral flow, whereas a distinct CB typically emerges later, most often during adolescence (Padget, 1948; Scheel et al., 2000; Schöning & Hartig, 1996). Pubertal increases in blood pressure and cardiac output, evolving flow distribution, and increased arterial stiffness generate complex branch hemodynamics, low‐oscillatory wall shear stress (WSS), and secondary flows at the ICA origin (Agbaje, 2022; Chistiakov et al., 2017; Jourdan et al., 2005; Ku et al., 1985; Lee et al., 2008; Urbina et al., 2010; Zarins et al., 1983; Zhou et al., 2014). These cues drive outward remodeling, producing a focal dilatation in many but not all individuals; accordingly, the CB reflects mechanoadaptive remodeling rather than a fixed congenital blueprint (Chien, 2007; Davies, 1995; Gijsen et al., 1999; Humphrey, 2021; Seong et al., 2005; Seong et al., 2015). Interindividual variation in adult CB morphology follows from differences in bifurcation geometry, hemodynamic load, and wall material properties (Dajnowiec & Langille, 2007; Glagov et al., 1987; Humphrey, 2021; Ku et al., 1985; Lee et al., 2008; Reckelhoff, 2005; Zarins et al., 1983). Across adulthood, bifurcation configuration continues to evolve, while CB dimensions show only weak associations with age or body mass index in disease‐free cohorts, supporting a dominant role for local vascular factors (Fazaldad et al., 2025; Seong et al., 2015).

### 3.2 Ontogeny of the carotid sinus baroreceptor field

CS baroreceptor terminals represent peripheral endings of sensory neurons from the petrosal (glossopharyngeal) ganglion that innervate the bifurcation region (Davies, 1995; Padget, 1948; Porzionato et al., 2019). These sensory neurons derive from epibranchial placodes, whereas neural crest supplies periarterial smooth muscle/pericytes and Schwann cells in this territory, not the sensory neurons themselves (Vermeiren et al., 2020; Wang et al., 2020). These sensory neurons extend peripheral axons into the adventitia of the developing arteries, where they form mechanosensitive endings that respond to wall stretch (D'Amico‐Martel & Noden, 1983; Hering, 1923; Le Douarin & Kalcheim, 1999; Schlosser, 2005; Toorop et al., 2013). The CS baroreceptor field differentiates during fetal and early postnatal life (Bamford et al., 1999; Blanco et al., 1988; Mazursky et al., 1998; Segar, 1997), with survival/maturation dependent on brain‐derived neurotrophic factor (BDNF)–tropomyosin receptor kinase B (TrkB) signaling and mechanosensitivity mediated by PIEZO1/PIEZO2, programs not contingent on CB geometry (Brady et al., 1999; Graham & Shimeld, 2013). Human mapping places the highest density of endings along the medial wall of the proximal ICA near the bifurcation, which explains the spatial colocalization with the CB without implying causation (Toorop et al., 2013). Thus, CS distribution may extend beyond a visible CB, and prominent CB geometry may occur in regions with relatively sparse baroreceptor innervation.

### 3.3 Modifiers: Sex, hormones, and genetics

Men generally have larger carotid luminal and carotid bulb diameters than women, a difference that may contribute to sex‐related variation in local bifurcation hemodynamics (Krejza et al., 2006; Schulz & Rothwell, 2001b; Tan et al., 2021). The hormonal milieu likely modulates this remodeling response: androgens are associated with greater outward vascular remodeling under hemodynamic load, whereas estrogens exert relative protective effects by limiting maladaptive smooth muscle proliferation and supporting endothelial stability (Cai et al., 2011; Chignalia et al., 2012; Hogg et al., 2012; Li et al., 1999; Song et al., 1998). Consistent with these combined geometric and hormonal influences, men exhibit a higher overall burden of carotid atherosclerosis and more frequent high‐risk plaque features, albeit with substantial interindividual overlap (van Dam‐Nolen et al., 2023). Genetic factors further modify susceptibility to carotid remodeling. Twin and family studies demonstrate heritability of carotid diameter and arterial stiffness, indicating that intrinsic vessel wall properties influence how similar macroscopic hemodynamic exposures translate into variable carotid bulb morphology across individuals (Bella et al., 2013; Bijari et al., 2014; Cecelja et al., 2018; Cui et al., 2019; Humphrey, 2008; Juo et al., 2005; Kuipers et al., 2019).

### 3.4 Clinical implications of developmental timing

Because the CB commonly emerges during adolescence, it can establish regions of low/oscillatory WSS decades before clinical disease, creating a durable geometric substrate for later atherosclerosis (Markl et al., 2010; Seong et al., 2015). This supports a two‐stage model (Frangos et al., 1999): Stage 1 (adolescence), growth‐associated hemodynamics sculpt CB geometry and focal adverse WSS, priming endothelium for lesion initiation, largely independently of traditional risk factors (Markl et al., 2010; Seong et al., 2015); Stage 2 (adulthood), systemic risks (e.g., elevated low‐density lipoprotein [LDL] cholesterol, oxidative stress, inflammatory activation) drive plaque formation and progression within pre‐existing geometric vulnerability (Gidding et al., 2016; Gidding et al., 2022). Accordingly, CB geometry influences where plaques form, while systemic exposures influence whether and how rapidly disease develops (Koskinas et al., 2013; Wentzel et al., 2012). This framework is consistent with plaque predilection for adverse‐flow regions (often the outer ICA wall where shear is lowest) and with wide interindividual variability in onset despite similar substrates (Gidding et al., 2016; Koskinen et al., 2023; Ku et al., 1985; Markl et al., 2010). Against this developmental background, we next examine how the distribution of baroreceptors at the carotid bifurcation relates to and diverges from luminal morphology.

## 4 WHY THE DISTINCTION MATTERS

Assuming that imaging‐visible bulb dilation marks baroreceptor location can misdirect CS massage, anesthetic infiltration, and CS‐targeted interventions (Iliescu et al., 2014; Kansal et al., 2016; Tordoir et al., 2007). The variable relationship between geometric dilatation and baroreceptor density explains why some patients exhibit profound hemodynamic instability with minimal manipulation, while others with prominent bulbs show little reactivity, a dissociation incomprehensible if the two entities are treated as equivalent (Gupta et al., 2006; Mylonas et al., 2013; Qureshi et al., 1999; Suh et al., 2012). Moreover, research reporting “carotid sinus” responses without distinguishing between geometric and functional anatomy undermines the systematic synthesis of findings and perpetuates terminological confusion across the literature.

## 5 SCOPE AND STRUCTURE OF THIS REVIEW

This review aims to deconstruct the historical conflation of the CB and CS by providing separate, rigorous characterizations of CB geometry and hemodynamics and CS neurohistological baroreceptor architecture, and by examining the clinical consequences of their frequent mislabeling. A targeted, non‐systematic appraisal of the anatomical, physiological, hemodynamic, and clinical literature addressing the CB and CS was conducted. Relevant literature was identified primarily through searches of PubMed/MEDLINE, Web of Science, and Google Scholar, covering sources from the mid‐nineteenth century to recent publications, using combinations of terms related to the carotid bifurcation, CB, CS, baroreceptors, baroreflex physiology, hemodynamics, and atherosclerosis. Seminal historical studies (e.g., Czermak, Hering) and classical anatomical texts (including *Gray's Anatomy*, Paturet, and Hollinshead) were intentionally included to document the evolution and persistence of terminology. Sources were selected based on their relevance to vascular geometry, neurohistological baroreceptor architecture, physiological function, or clinical implications, without formal quality scoring or quantitative synthesis, as the primary aim was conceptual clarification rather than systematic evidence aggregation.

## 6 A HISTORICAL PERSPECTIVE ON TERMINOLOGICAL CONFUSION

The current ambiguity surrounding the terms “carotid bulb” and “carotid sinus” is not a recent development but rather a legacy of over a century of evolving physiological understanding. The roots of the confusion lie in early observations that correctly linked the carotid bifurcation to profound cardiovascular reflexes but misidentified the mechanistic basis, conflating a visible geometric landmark with an invisible field of sensory receptors.

### 6.1 From káros to the carotid pressure test

The term “carotid” derives from Greek káros (κάρος, “stupor/sleep”), reflecting ancient reports that compression of the neck arteries induced stupor (Mohr, 2022; Munster et al., 2016; Robicsek et al., 2004). Rufus of Ephesus records that the ancients called these vessels καρωτίδες (karōtides, “stupor arteries”) because pressing them made the animal “sleepy and lose its voice” (Bujalkova, 2011). However, he clarified that the effect resulted from pressure on adjacent nerves, an observation documented in his “On the Names of the Parts of the Human Body” (Onomastikon) (Bujalkova, 2011). This ancient recognition that the phenomenon involved “adjacent nerves” anticipated the search for neural mechanisms, which would lead to Czermak's erroneous hypothesis of vagal compression.

Modern investigation of the specific reflexes of this region began in the 19th century (Czermák, 1865; Scheffers et al., 2010; Zimmer, 2004). In 1865, Czech physiologist Johann Nepomuk Czermak (Jan Czermak) published a self‐experiment demonstrating that pressure over the carotid artery at the neck could dramatically slow the heart rate, reducing pulse frequency by half or more (Czermák, 1865). By applying firm pressure to the right side of his neck over the carotid pulsation, where the artery emerged beneath the sternocleidomastoid muscle, he induced a dramatic slowing of his heart rate, with pulse frequency decreasing by half or more, which he documented with pulse tracings from Marey's sphygmograph (Figure 2) (Czermák, 1865). The publication included three woodcut curves showing the characteristic response: a prolonged diastolic pause followed by a stronger, larger pulse beat (Czermák, 1865). Czermak termed this “Vagusdruckversuch” (“vagus pressure test”) (Czermák, 1865).

**FIGURE 2:**
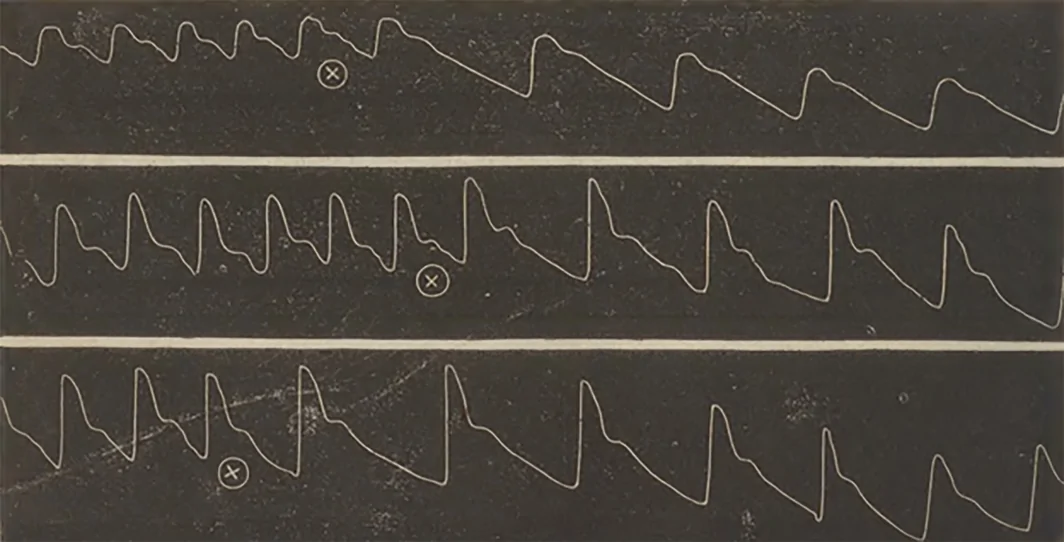
Czermak's original sphygmograph tracings (1865). Three radial pulse curves recorded by Johann Nepomuk Czermak during his self‐experiments on right‐sided carotid compression (“Vagusdruckversuch”). Each tracing shows a prolonged diastolic pause and a reduced pulse frequency following external pressure applied to the carotid artery, a finding Czermak attributed to direct compression of the vagus nerve. This interpretation, though incorrect, established the historical conflation between the carotid bulb and carotid sinus. Reproduced from Czermák (1865), woodcut sphygmographic pulse curves obtained during carotid‐region pressure. Public domain.

### 6.2 The roots of conflation: Czermak's misinterpretation

Czermak's observation was correct, but his interpretation of the mechanism was incorrect; this error planted the seed of the terminological confusion that persists today. He attributed the bradycardia to direct mechanical compression of the vagus nerve, which runs in the carotid sheath adjacent to the CCA and ICA (Czermák, 1865). Specifically, he hypothesized that external pressure on the neck pushed the “bulb‐like dilatation” of the carotid artery against the vagus nerve, directly stimulating its cardioinhibitory fibers (Czermák, 1865). This was the foundational misattribution: it created a direct mechanistic link between the observable geometric bulge and the physiological response.

Critically, Czermak reported that the maneuver succeeded only on the right side of his own neck and could not be reproduced in other subjects (Czermák, 1865). This observation, though Czermak did not recognize its significance, provided early evidence that the reflexogenic zone exhibits marked interindividual and bilateral variability. The reflex response did not correlate simply with the presence or prominence of the carotid bulge, but rather with the highly variable distribution and accessibility of underlying sensory receptors. Hering's discovery would later explain this variability, that the reflex originates from specialized nerve endings within the arterial wall, not from the geometric features Czermak was palpating.

### 6.3 Hering's correction and the persistent linguistic mismatch

Czermak's hypothesis held sway for more than 50 years until Heinrich Ewald Hering's meticulous work systematically dismantled it. Inspired by his teacher's observation that the glossopharyngeal nerve might function as a “depressor nerve,” Hering conducted a series of elegant experiments in dogs and rabbits beginning in the early 1920s (Hering, 1925; Shoja et al., 2019). He demonstrated that direct manipulation of the exposed vagus nerve often failed to produce the profound bradycardia seen with carotid pressure (Hering, 1923, 1925; Shoja et al., 2019). In contrast, very gentle pressure applied directly to the arterial wall at the carotid bifurcation consistently elicited the reflex (Hering, 1923, 1925; Shoja et al., 2019). Crucially, surgical denervation of the carotid sinus region, dissection, and removal of tissues within the bifurcation completely abolished both the heart‐inhibitory and vasodepressor reflexes (Hering, 1923, 1925; Shoja et al., 2019).

In his landmark 1925 paper, “Ueber die Wand des Sinus caroticus als Reizempfänger und den Sinusnerv als zentripetale Bahn für die Sinusreflexe” (“About the wall of the carotid sinus as a stimulus‐receiver and the sinus nerve as a centripetal pathway for the sinus reflexes”), Hering definitively proved that the effects of Czermak's maneuver were “not the result of pressure on the vagal nerve and the mechanical irritation of its heart inhibitory fibers, but due to a reflex from the carotid sinus, which is located at the origin of the internal carotid artery” (Hering, 1925; Shoja et al., 2019). He identified that the stimulus originated from mechanoreceptors (baroreceptors) within the arterial wall itself and that afferent signals traveled to the brainstem via a specific branch of the glossopharyngeal nerve (CN IX), the first branch visible after its exit from the skull base, which he named the “Sinusnerv” (sinus nerve, now known as Hering's nerve or the carotid sinus nerve, CSN) (Hering, 1923, 1925; Shoja et al., 2019). He correctly renamed the maneuver the “Karotisdruckversuch” (“carotid pressure test”) to reflect the arterial, rather than vagal, origin of the reflex (Hering, 1923).

Although Hering correctly identified the physiological mechanism and appropriately named the reflexogenic area the “carotid sinus,” the damage to terminology had already been done. The term “sinus” remained inextricably linked in the clinical mind to the most obvious and palpable anatomical landmark in the region: the “bulb.” Because the invisible field of baroreceptors was in the general vicinity of the visible dilatation, the two terms slid into synonymous usage. The ease of seeing and feeling the CB led to its name being co‐opted for the reflex, cementing a century of confusion in the medical lexicon.

The very etymology of “sinus” compounded the problem. Derived from Latin for “bay,” “pocket,” or “hollow,” the term is used throughout anatomy to describe dilated spaces, from paranasal sinuses to venous sinuses. When Hering chose “carotid sinus” to label the reflexogenic area, he inadvertently selected a word that also served as a perfect geometric descriptor for the bulbous dilatation itself. For early 20th‐century clinicians, “carotid sinus” evoked both the newly described reflex and the pre‐existing concept of an anatomical dilation. This linguistic overlap reinforced the conflation: unlike a more specific eponym, “sinus” carried strong anatomical connotations of dilated space, making it natural to map Hering's physiological concept directly onto the most obvious geometric landmark.

## 7 THE CAROTID BULB: A MATTER OF VASCULAR GEOMETRY AND HEMODYNAMICS

### 7.1 Gross anatomy and morphometric variability

Reported CB morphology varies substantially across individuals and across studies, in part because investigators use different landmarks and thresholds when describing the “bulb” region (Bijari et al., 2014; Fazaldad et al., 2025; Gregg et al., 2018; Kamenskiy et al., 2012; Schulz & Rothwell, 2001a; West et al., 2018). The CB is therefore marked by profound interindividual variability in both its location and dimensions, and, critically, its reported presence is inconsistent, being identifiable in some individuals and not in others (Hu et al., 2018; Morimoto et al., 1990; Uchino et al., 2011; Yoshida & Ota, 2016).

#### 7.1.1 Operational definitions for interpreting prior studies

A recurring mislabeling bias is that many studies use “carotid sinus” to denote an imaging‐defined dilatation, and then interpret geometric measurements as if they mapped the baroreceptor field (Baz et al., 2022; Fazaldad et al., 2025; Finucane et al., 2016; Querry et al., 2001; West et al., 2018). More consequentially, this framing invites invalid inference because baroreceptor distribution is a neurohistological property that cannot be deduced from luminal contour alone (Seong et al., 2015).

Critical observers have explicitly recognized the terminological chaos surrounding the CB. Toole & Castaldo, in their comprehensive analysis of carotid circulation nomenclature, documented that inconsistent definitions of both the location and dimensions of these structures have compounded the confusion in the literature (Toole & Castaldo, 1994). They identified a striking disciplinary divide: imaging specialists using ultrasound tend to identify the CB as the widening of the terminal CCA (Toole & Castaldo, 1994). At the same time, those in radiology and surgery more commonly treat it as equivalent to a dilated proximal ICA, in which baroreceptors are presumed to reside (Toole & Castaldo, 1994). This professional fragmentation reflects not merely different perspectives on the same structure but fundamentally different conceptual frameworks: one focusing on geometric landmarks visible on imaging, the other assuming functional equivalence with the baroreceptor zone. Equally important, they noted that “carotid bulb” is absent from standardized anatomical nomenclature and much of the classical anatomy literature (FIPAT, 2019; Sinnatamby & Last, 2011; Standring et al., 2020). Thus, a term with no official anatomical definition is nevertheless widely used in imaging reports, surgical descriptions, and research, thereby perpetuating inconsistency between formal nomenclature and clinical usage (Gregg et al., 2018; Nicholls et al., 1989; Sajedi et al., 2017). Finally, while their analysis is careful, adopting Dorland's definition still links the CS to a dilation (Dorland, 1925). Abandonment of the term “carotid bulb” has been advocated; in ultrasound reporting, its elimination in favor of precise vessel‐segment descriptors has been recommended (Thornton et al., 2007; Toole & Castaldo, 1994). While we acknowledge this concern, we argue that elimination is neither necessary nor desirable.

Major morphometric studies from the past two decades label the visible dilatation as the “carotid sinus” (Baz et al., 2022; Fazaldad et al., 2025; Nagargoje & Gupta, 2020; Seong et al., 2015; West et al., 2018). While valuable for characterizing vascular geometry, these studies perpetuate terminological ambiguity by conflating a macroscopic structural feature with a microscopic neurohistological entity. Their measurements indicate where the arterial wall dilates rather than where baroreceptors are concentrated. Thornton et al. (2007) explicitly studied the “carotid bulb” as a purely geometric entity, avoiding the term “sinus” and emphasizing that dilatation is independent of baroreceptor function (Thornton et al., 2007). They noted that anatomical textbooks locate the CB at different arterial segments, ICA, CCA, or the entire bifurcation, reflecting the underlying reality that there is no single “correct” location because the CB is not a defined anatomical structure but a variable geometric feature (Thornton et al., 2007). They also correctly stated that the CB may house the CS; thus, they explicitly distinguished the two entities while acknowledging their potential overlap (Thornton et al., 2007).

#### 7.1.2 Positional variability of the carotid bulb

Across imaging and cadaveric studies, the CB behaves as a geometric variant rather than a fixed site. Although classical teaching places the CB at the ICA origin, this is simply the most frequent pattern rather than a universal rule. Morphologically, four recurring configurations capture most observations. Type I (classical) is a focal dilatation at the proximal ICA just above the flow divider and is the predominant form (Baz et al., 2022; Fazaldad et al., 2025; West et al., 2018). Type II (trifurcation) involves the distal CCA and the origins of both the ICA/external carotid artery (ECA) and is uncommon (Fazaldad et al., 2025; West et al., 2018). Type III (prebifurcation) is limited to the distal CCA and exhibits the widest interstudy variation, reflecting differing thresholds for what constitutes “significant” dilatation, a problem long noted in critiques of dimensional definitions (Fazaldad et al., 2025; West et al., 2018). Type IV (ECA‐origin) is rare. Thornton et al. provide the clearest picture by abandoning assumptions about where the CB “should” be and scanning the entire bifurcation with duplex ultrasound (Thornton et al., 2007). They found dilation limited to the ICA in about half of carotid systems; combined patterns involving ICA + ECA or ICA + CCA in a meaningful minority of cases; occasional cases confined to the CCA or to the ECA alone; and, importantly, nearly one in 10 systems with no discernible CB (Thornton et al., 2007). These data confirm that CB location can be ICA‐only, CCA‐only, ECA‐only, any combination of the three, or absent altogether. Taken together, the literature supports a simple conclusion: the CB is a macromorphologic dilation whose position varies across the bifurcation complex. This variability helps explain disciplinary disagreement about “where the bulb is.” It underscores the merit of reporting with precise vessel‐and‐segment descriptors (proximal/distal ICA, distal CCA, ECA origin) rather than relying on the ambiguous umbrella term “carotid bulb.”

#### 7.1.3 Bilateral asymmetry

The CB's variability extends beyond its longitudinal location to include bilateral asymmetry within the same individual. Furukawa et al., in a cadaveric study of 100 specimens, reported right–left asymmetry in 54% of cases, indicating that more than half of individuals exhibit bilateral morphological differences at the bifurcation. Similarly, West et al. found asymmetry in 34.1% of their cohort. This asymmetry encompasses differences in CB location (e.g., Type I on the right, Type III on the left), extent (a longer CB on one side), and even presence versus absence (a CB present unilaterally) (West et al., 2018). This likely reflects local, side‐specific hemodynamics and has practical implications: clinicians cannot assume that findings on one side predict the contralateral anatomy, and surgical or endovascular interventions must account for side‐specific geometric variations. An uncommon example of bilateral symmetry with distal placement of the CBs along the ICAs is shown in Figure 3. In contrast, Figure 4 illustrates side‐to‐side variation, a right‐sided CB confined to the proximal ICA and a broader left‐sided CB extending from the distal CCA into the proximal ICA. Atheromatous plaques may be present at the carotid bifurcation and along the ICA, but these plaque‐related contour changes should not be interpreted as defining CB morphology.

**FIGURE 3:**
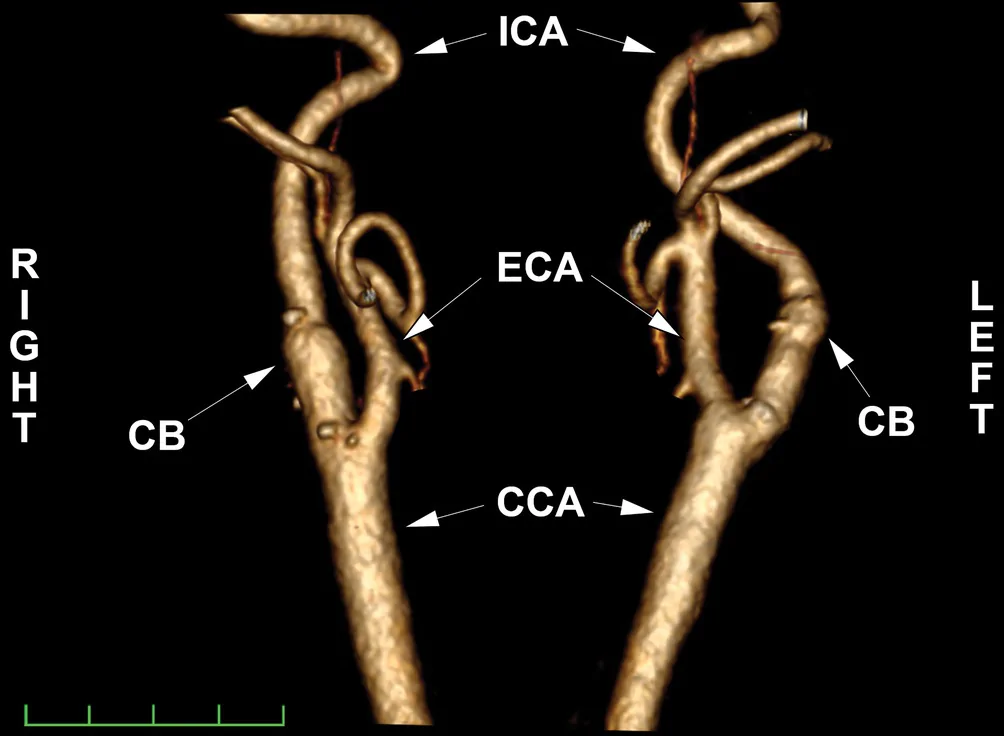
Original three‐dimensional reconstruction demonstrating bilateral distal internal carotid artery (ICA) bulbs, an uncommon morphological variant, with carotid bulbs (CBs) positioned distally along the ICAs, superior to the common levels. CCA, common carotid artery; ECA, external carotid artery. Scale bar, 5 mm.

**FIGURE 4:**
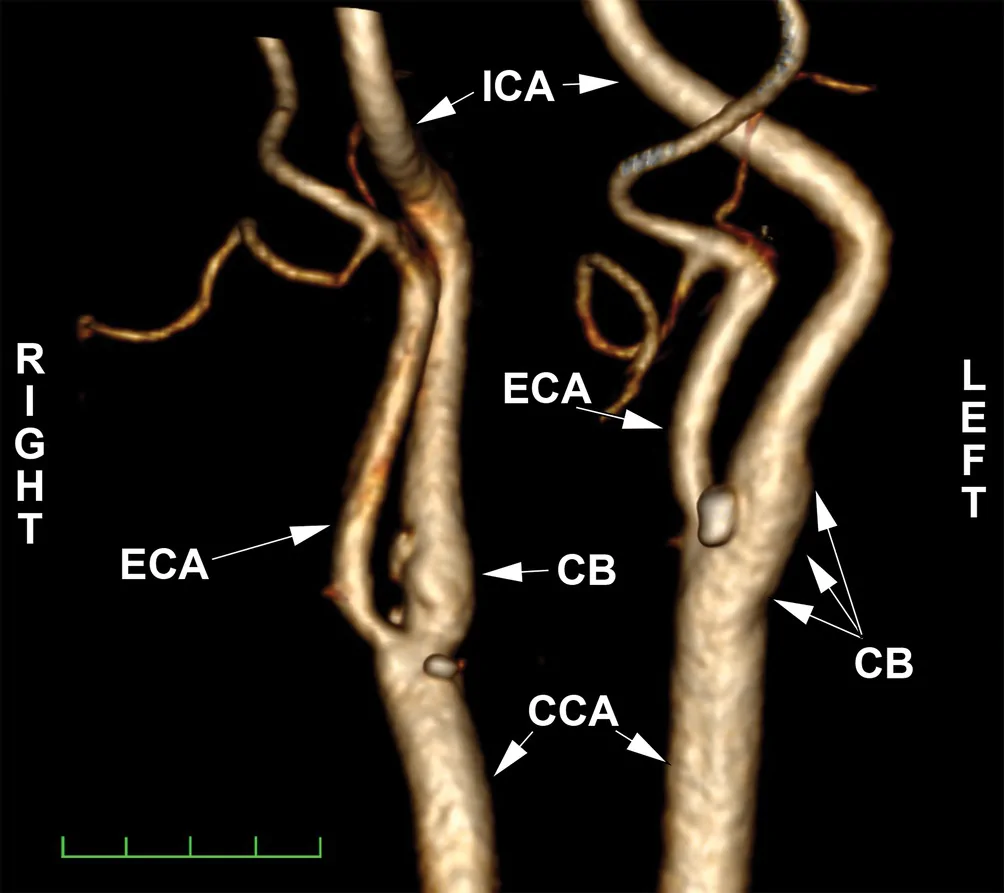
Original three‐dimensional reconstruction of asymmetric carotid bulb (CB) morphology with distinct bilateral configurations. On the right side, the CB is confined to the proximal internal carotid artery (ICA), representing the classical pattern. On the left side, the CB extends from the distal common carotid artery (CCA) into the proximal ICA, forming a broader, more prominent dilation. ECA, external carotid artery. Scale bar, 5 mm.

#### 7.1.4 Vertebral level variability

The vertical position of the carotid bifurcation, and thus the CB when present, varies widely. Although classically taught at the superior border of the thyroid cartilage (C3–C4) (Paturet, 1951; Standring et al., 2020), contemporary morphometry refines this view. In a cohort of 294 bifurcations, peak frequencies were observed at C3 (27%), C3/C4 (26%), and C4 (26%), accounting for nearly 80% of cases. Less common levels included C2 (8%), C4/C5 (6%), C5 (4%), and C5/C6 (3%), confirming that both high (C1–C2) and low (C5–C6) bifurcations occur but are relatively infrequent (Manta et al., 2024). Bilateral height symmetry was present in approximately 52%, with the remainder showing right–left asymmetry (Manta et al., 2024). Importantly, statistically significant correlations were found between sex and both sides and vertebral levels, suggesting that vertical carotid topography is biologically structured rather than random (Manta et al., 2024). These findings have surgical relevance: higher bifurcations (C2–C3) pose greater difficulty for exposure and nerve preservation, while lower bifurcations (C5 or below) complicate proximal vascular control.

#### 7.1.5 Dimensional characteristics

When the CB is present, its dimensions vary significantly across individuals and between sexes. Fazaldad et al. documented mean CB length (the longitudinal extent of dilatation) of 12.7 mm in men and 11.8 mm in women, with statistically significant sex differences (*p* < 0.05) (Fazaldad et al., 2025). Baz et al. reported a mean CB length of 9.99 ± 2.22 mm, again with males exhibiting longer CBs than females (Baz et al., 2022). These values represent population averages; individual measurements range from less than 5 mm to over 20 mm, illustrating the substantial interindividual heterogeneity.

Vessel diameters at the bifurcation also vary by sex and age. Baz et al. measured mean diameters of 7.39 ± 1.04 mm for the CCA, 6.71 ± 1.49 mm for the ICA, and 4.27 ± 0.75 mm for the ECA in disease‐free arteries, with males demonstrating larger diameters across all segments (Baz et al., 2022). Importantly, Fazaldad et al. found that the maximal diameter occurred at the CCA level (distal CCA or CB proper) in 99% of cases, not at the ICA or ECA, confirming that the CB, when present, typically represents the widest point in the carotid system at the bifurcation (Fazaldad et al., 2025).

A recent report describes a fenestrated carotid bifurcation, in which a longitudinal division extended from the distal CCA into the proximal ICA (Rusu et al., 2025). In this configuration, no actual CB was identifiable; the segment typically associated with focal dilation appeared to be replaced by two parallel channels that rejoined distally (Rusu et al., 2025). This morphology exemplifies the non‐obligatory nature of the CB and demonstrates that even significant deviations from the classic bifurcation form can occur without eliminating vascular continuity or flow. The case visually reinforces our central thesis: the CB is a geometric variant, not a fixed anatomic constant, and its presence or contour can be profoundly altered, or absent, without implying any change in the functional baroreceptor field of the CS (Figure 5).

**FIGURE 5:**
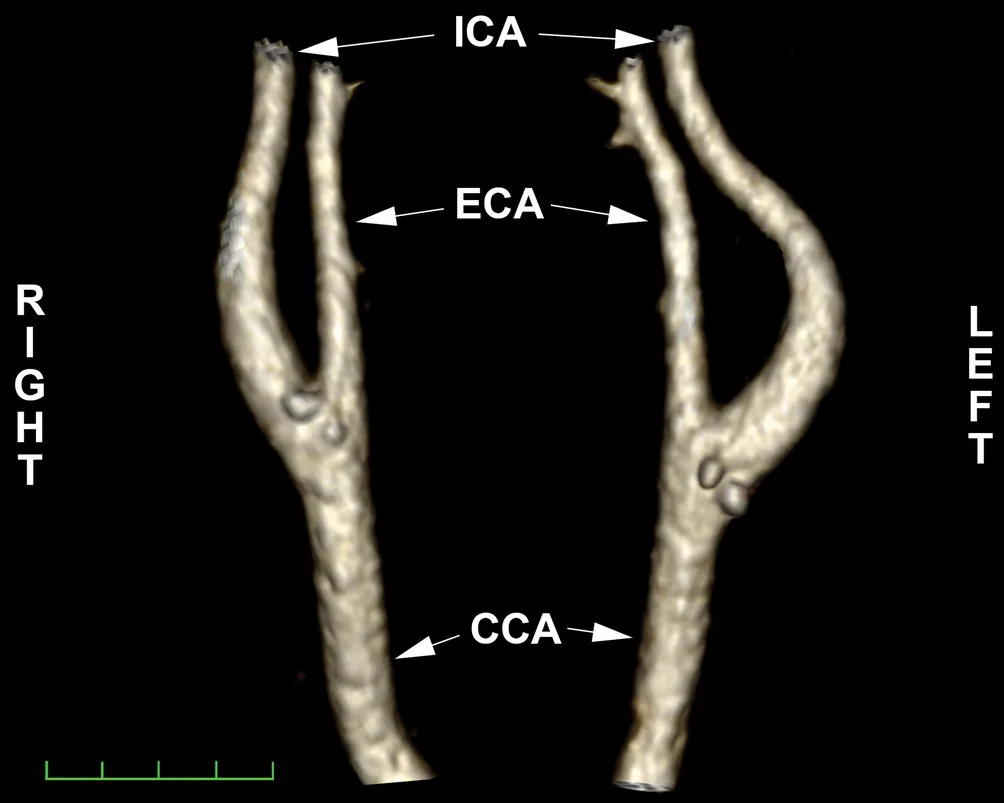
Original three‐dimensional CT angiographic reconstruction demonstrating the absence of the carotid bulb (CB) bilaterally. Anterior view showing carotid bifurcations without focal luminal dilatation. Both sides exhibit smooth CCA‐to‐ICA transitions, without the typical fusiform expansion that defines the CB. ECA, external carotid artery; CCA, common carotid artery; ICA, internal carotid artery. Scale bar, 5 mm.

### 7.2 The bulb as a hemodynamic locus

The primary clinical significance of the CB's geometry lies in its profound impact on local blood flow dynamics (Milner et al., 1998; Steinman, 2002). The CB represents a biomechanical “hot spot” where arterial architecture creates uniquely adverse hemodynamic conditions that directly predispose to endothelial dysfunction and atherosclerotic disease (Malek et al., 1999; Zarins et al., 1983). Understanding these flow disturbances is essential for comprehending why the carotid bifurcation, and specifically the CB, is one of the most common sites for extracranial atherosclerosis and stroke‐producing stenosis (Shaaban & Duerinckx, 2000).

#### 7.2.1 Geometric foundations of disturbed flow

Disturbed hemodynamics at the CB arise from the interplay of abrupt luminal expansion, bifurcation‐related flow division, and branch angulation/curvature (Ku et al., 1985; Zarins et al., 1983). First, the abrupt expansion from the CCA into the dilated CB (“flaring”) causes rapid deceleration, separation, and recirculation along the outer wall of the CB (Bijari et al., 2014; Ku et al., 1985). Second, bifurcation and flow division impose asymmetric outflow: the low‐resistance ICA carries continuous diastolic flow, whereas the ECA perfuses a higher‐resistance bed, shaping distinct velocity waveforms and downstream boundary conditions that feed back on near‐bifurcation flow (Lee et al., 2008). Third, the angulation and curvature of the bifurcation introduce secondary flows and helical patterns that further skew the WSS distribution (Harloff et al., 2009; Milner et al., 1998). Together, these geometric factors generate low and oscillatory wall shear, separation, and flow reversal, hallmarks of “disturbed flow” that are strongly linked to endothelial dysfunction and atheroprone biology at the CB (Ku et al., 1985; Zarins et al., 1983). Notably, in physiologic (non‐stenotic) carotids, this disturbance is typically complex but not fully turbulent, with true turbulence mainly appearing downstream of significant stenoses (Goddi et al., 2017). Because CB topology varies widely between individuals, the magnitude and extent of disturbed‐shear exposure also differ substantially, even under similar inflow conditions (Bijari et al., 2014; Lee et al., 2008). Modern computational fluid dynamics (CFD) and 4D‐flow MRI studies confirm that geometry is a dominant determinant of where and how much disturbed flow occurs at the carotid bifurcation (Lee et al., 2008; Ngo et al., 2019). Accordingly, interindividual variation in bulb presence/size and bifurcation angulation helps explain why some carotids exhibit large recirculation zones while others remain comparatively stable (Abhilash et al., 2024; Steinke et al., 1990).

#### 7.2.2 Flow separation and the recirculation zone

As blood enters the expanded CB from the CCA, the abrupt luminal widening causes flow deceleration and separation along the outer wall of the ICA bulge (Ngo et al., 2021; Nicholls et al., 1989). This creates a recirculation zone characterized by low velocity, flow reversal during diastole, and vortex formation, a hemodynamic environment fundamentally distinct from the laminar, unidirectional flow in straight arterial segments (Compagne et al., 2019; Ngo et al., 2021; Wild et al., 2024). CFD modeling and phase‐contrast MRI studies consistently demonstrate that this zone of disturbed flow colocalizes precisely with the anatomical sites of preferential atherosclerotic plaque development: the posterolateral wall of the ICA bulb (Cho et al., 2024; Ren et al., 2023; Yang et al., 2025).

The pathological significance of this recirculation zone lies in its effects on WSS, the tangential frictional force exerted by flowing blood on the endothelium (Gallo et al., 2018; Zhou et al., 2023). Within regions of flow separation, WSS not only drops to atherogenic levels but also oscillates in direction throughout the cardiac cycle, creating the mechanical stimulus that drives endothelial dysfunction and plaque formation (Gallo et al., 2018; Strecker et al., 2020; Weinberg, 2022). This hemodynamic consequence of CB geometry is entirely independent of CS baroreceptor function, which responds to circumferential wall strain induced by transmural pressure, not to tangential forces from blood flow or WSS (Dessalles et al., 2021; Katoh, 2023; Suarez‐Roca et al., 2021; Tu et al., 2019).

#### 7.2.3 Hemodynamic forces and atherogenesis at the carotid bulb

The CB's adverse hemodynamic environment is characterized by three interrelated parameters (Table 2): chronically low WSS magnitude, oscillatory directional reversal, and prolonged particle residence time (Chen et al., 2024; Cheng et al., 2025; Li et al., 2022; Roux et al., 2020). In atheroprotective laminar‐flow segments, WSS typically exceeds ~1.5–2.0 Pa, whereas within the CB recirculation zone, WSS frequently falls below ~0.4 Pa, a range associated with endothelial dysfunction, pro‐inflammatory signaling (e.g., nuclear factor κB, NF‐κB), and increased LDL permeability (Chen et al., 2024; Kashyap et al., 2022; Zhou et al., 2014). Directional instability amplifies these effects: systolic forward flow is followed by diastolic flow reversal, producing oscillatory WSS; correspondingly, the oscillatory shear index (OSI) often reaches >0.3 in the outer CB, exceeding commonly used atherogenic thresholds (~0.15–0.2) (Markl et al., 2010; Moerman et al., 2021; Patalano, 2023; Zalud et al., 2023). An integrated consequence of low/oscillatory shear is increased relative residence time (RRT), which peaks along the CB outer wall and colocalizes with early lesions in young adults and established plaques in older cohorts (Chen et al., 2025; Peiffer et al., 2013; Riccardello Jr. et al., 2018; Trenti et al., 2022). Prolonged near‐wall dwell time enhances LDL retention/oxidation and facilitates leukocyte adhesion and transmigration, accelerating atherogenesis (Chappell et al., 1998; Islam, 2020; Mundi et al., 2018; Owen et al., 2021). Critically, these luminal shear‐mediated mechanisms are independent of baroreceptor function: baroreceptors primarily transduce circumferential wall strain from transmural pressure rather than shear at the endothelial surface, allowing dissociation between CB atherogenic flow disturbance and baroreflex sensitivity in vivo (Chapleau et al., 2001; Knoche et al., 1977; Salah et al., 2025; Toorop et al., 2009; Toorop et al., 2013).

**TABLE 2:** Hemodynamic metrics at the carotid bulb and their putative atherogenic links.

Hemodynamic parameter	Definition	Typical state in CB outer wall	Atherogenic mechanisms	References
Wall shear stress (WSS)	Tangential frictional force of flowing blood on the endothelial surface (Pa or dynes/cm^2^)	Low (<0.4 Pa/4 dynes/cm^2^)	Downregulates eNOS and KLF2; upregulates NF‐κB; increases endothelial permeability to LDL; promotes pro‐inflammatory phenotype; induces adhesion molecule expression	Ku et al. (1985), Dai et al. (2004), Chiu and Chien (2011), and Gimbrone and Garcia‐Cardena (2016)
Oscillatory shear index (OSI)	Measure of temporal change in WSS direction over cardiac cycle (dimensionless, 0–0.5)	High (>0.3)	Maximally activates inflammatory signaling; induces endothelial‐to‐mesenchymal transition; impairs endothelial repair; amplifies oxidative stress	Himburg et al. (2004), Chatzizisis et al. (2007), and Feaver et al. (2013)
Relative residence time (RRT)	Time blood particles remain near the vessel wall, inversely related to WSS magnitude and directionality	High (>10–20 arbitrary units)	Prolongs exposure to atherogenic lipoproteins; enhances LDL oxidation; increases monocyte adhesion and transmigration; facilitates platelet activation	Himburg et al. (2004), Gallo et al. (2018), and Owen et al. (2021)
Time‐averaged WSS	Mean magnitude of WSS over the entire cardiac cycle	Low (<0.5 Pa)	Sustained endothelial dysfunction; chronic pro‐inflammatory state; impaired nitric oxide bioavailability	Markl et al. (2010), Wentzel et al. (2012), and Peiffer et al. (2013)

Abbreviations: CB, carotid bulb; eNOS, endothelial nitric oxide synthase; KLF2, Krüppel‐like factor 2; LDL, low‐density lipoprotein; NF‐κB, nuclear factor κB; OSI, oscillatory shear index; RRT, relative residence time; WSS, wall shear stress.

We now turn from luminal morphology and flow‐related phenomena at the bifurcation to evidence defining the distribution of baroreceptors and reflexogenic tissue.

## 8 THE CAROTID SINUS: A NEUROHISTOLOGICAL AND PHYSIOLOGICAL CONSTRUCT

### 8.1 Histological mapping of the baroreceptor field

The baroreceptors that constitute the CS are specialized mechanosensitive nerve endings located predominantly within the tunica adventitia, the outermost layer of the arterial wall (Andani & Khan, 2025; Toorop et al., 2013). The baroreceptive region exhibits structural adaptations that enhance mechanotransduction, including a thinned tunica media, reduced intima, and increased elastic fiber content compared to adjacent arterial segments (Bryant et al., 2023; Porzionato et al., 2019). This more compliant wall architecture amplifies deformation in response to transmural pressure changes, thereby enhancing the mechanical stimulus delivered to the embedded nerve terminals and increasing baroreceptor sensitivity (Chapleau et al., 2001).

While early anatomists associated the CS with the entire carotid dilatation, modern histological mapping techniques have provided a much more refined understanding of the baroreceptor distribution (Porzionato et al., 2019; Shoja et al., 2019; Toorop et al., 2013). Immunohistochemical studies, which utilize antibodies to stain for specific neural markers such as vesicular glutamate transporter 2 (VGLUT2) and protein gene product 9.5 (PGP 9.5), have visualized the baroreceptor field with high precision (Toorop et al., 2013; Yokoyama et al., 2020). These studies have conclusively shown that baroreceptors are not uniformly distributed throughout the CB (Toorop et al., 2013). Instead, the nerve endings are most densely concentrated along the medial wall of the proximal first centimeter of the ICA (Toorop et al., 2013). While some sparse neural tissue can be found in the distal CCA and the origin of the ECA, the functional core of the CS resides in this specific region of the ICA (Struthoff et al., 2024; Toorop et al., 2013).

Functionally, the baroreceptor population is heterogeneous, comprising at least two distinct fiber types that encode different aspects of the pressure signal (Brown, 1980; Porzionato et al., 2019). Type 1 (dynamic) receptors, associated with large, myelinated A‐fibers, are sensitive to the rate of pressure change (Andani & Khan, 2025; Brown et al., 1978; Porzionato et al., 2019). Type 2 (tonic) receptors, associated with smaller myelinated A‐fibers and unmyelinated C‐fibers, respond primarily to absolute steady‐state pressure (Andani & Khan, 2025; Brown et al., 1978; Porzionato et al., 2019). This dual‐receptor system provides the central nervous system with comprehensive information about both mean arterial pressure and its pulsatile dynamics (Brown, 1980).

In the absence of human series documenting congenital absence of CS baroreceptor endings, it is prudent to assume a potentially recruitable reflexogenic field, irrespective of CB geometry, and to reflect this in reporting and procedural planning.

### 8.2 Innervation and the afferent pathway of the baroreflex

CS baroreceptors are the peripheral mechanosensory terminals of the arterial baroreflex, providing afferent signals that stabilize arterial pressure beat by beat (Pirahanchi & Bordoni, 2025). Action potentials generated by wall stretch travel via the CSN, a branch of the glossopharyngeal nerve (CN IX) that typically arises a few millimeters distal to the jugular foramen (Porzionato et al., 2019; Shoja et al., 2019). The primary sensory neurons reside in the petrosal (inferior) ganglion of CN IX, with central axons projecting to the nucleus tractus solitarius (NTS) in the medulla, where baroreceptor afferents terminate in specific subnuclei distinct from chemoreceptor pathways (Porzionato et al., 2019). The CSN exhibits anatomical variability in its branching pattern and frequently communicates with the vagus nerve and the sympathetic trunk, forming an intercarotid plexus of mixed glossopharyngeal, vagal, and sympathetic fibers (Kikuta et al., 2019; Porzionato et al., 2019). This complex innervation pattern has clinical relevance for CS syndrome (CSS) surgery and explains variable responses to carotid manipulation, but does not alter the fundamental principle that baroreceptor afferents are sensory fibers responding to wall strain, not luminal flow (Chapleau et al., 2001; Timmers et al., 2003). Recent molecular studies have identified PIEZO1 and PIEZO2 mechanosensitive ion channels as critical mechanotransducers in arterial baroreceptor neurons; genetic disruption blunts baroreflex‐mediated bradycardia and blood pressure regulation (Machado et al., 1997; Zeng et al., 2018). These findings reinforce the CS as a specialized mechanosensory system that transduces circumferential wall strain into neural signals, operating independently of the hemodynamic forces (WSS, flow patterns) that characterize the CB.

## 9 SPATIAL DISSOCIATION AND THE FLAWED ASSUMPTION OF IDENTITY

As established, the CS baroreceptors are most densely packed on the medial wall of the proximal ICA (Porzionato et al., 2019; Toorop et al., 2013). In contrast, the hemodynamic forces that drive atherosclerosis, namely, low and oscillatory WSS, are most pronounced on the posterolateral wall of the CB, within the zone of flow separation (Gharahi et al., 2016; Ku et al., 1985; Zarins et al., 1983). This creates a fundamental spatial mismatch. The site of peak physiological function (baroreception) is often anatomically distinct from the site of peak pathological stimulus (atherogenesis).

This dissociation has profound implications. A large atherosclerotic plaque developing on the posterolateral wall of the CB, the most common location, may have minimal direct mechanical impact on the main baroreceptor field on the opposite wall. Conversely, a smaller, less stenotic plaque strategically located on the medial wall could significantly alter the mechanical properties of the baroreceptive arterial wall, potentially leading to profound baroreflex dysfunction. Importantly, conventional vascular imaging (CTA, ultrasound, angiography) visualizes CB geometry and plaque morphology but cannot assess baroreceptor field integrity or distribution, underscoring the inability to infer CS function from CB appearance alone (Brignole et al., 2018; Dittmar & Feldman, 2016).

This leads to a more refined understanding of the pathologies of the carotid bifurcation. It is not a single entity prone to “carotid sinus disease.” Instead, it is a complex region that can host two distinct, though potentially interacting, disease processes. The CB is the primary site of a structural disease, atherosclerosis, driven by local hemodynamics. The CS is the origin of a functional disease, CS hypersensitivity (CSH), driven by neuro‐reflexive dysregulation. Structural disease in the CB can influence functional disease in the CS, for example, by stiffening the arterial wall and altering baroreceptor stimulation, but they remain fundamentally different conditions. Treating them as a single entity obscures this crucial interaction. It limits a clinical understanding of patients who may present with both stroke risk from plaque in the CB and syncope risk from a hypersensitive CS.

## 10 CLINICAL AND SURGICAL IMPLICATIONS OF THE CAROTID BULB AND SINUS DISTINCTION

The failure to consistently differentiate between the CB and CS has direct consequences for clinical practice. Conflated terminology creates ambiguity in diagnostic imaging, obscures dual pathology in risk assessment, and increases procedural risks during carotid interventions. As neuromodulatory therapies targeting baroreceptors emerge, precise anatomical nomenclature becomes not merely academic but clinically essential.

### 10.1 Diagnostic ambiguity and risk assessment

In clinical practice, imprecise terminology compromises diagnostic clarity and risk stratification. When imaging reports describe “atherosclerotic plaque within the carotid sinus,” the interpretation is ambiguous: does this refer to plaque occupying the geometric space of the CB, or does it imply mechanical impingement on the baroreceptor field? Given the spatial dissociation between plaque location (typically posterolateral) and baroreceptor concentration (medial), this distinction is clinically significant. A patient's risk profile should encompass both embolic stroke risk from plaque burden and potential hemodynamic instability from baroreflex dysfunction, risks that may vary independently depending on plaque location and baroreceptor field integrity (Brignole et al., 2018; Kleindorfer et al., 2021).

Similarly, attributing CSS, a functional disorder characterized by exaggerated baroreflex responses causing syncope or presyncope, to “carotid bulb disease” is mechanistically imprecise. While arterial stiffening from atherosclerosis may modulate baroreceptor sensitivity, CSS fundamentally reflects dysregulation of the reflex arc (peripheral baroreceptor sensitivity, central processing, or efferent response), not structural vascular disease (Tea et al., 1996). CSS can occur with minimal atherosclerotic burden, and conversely, severe CB atherosclerosis can occur without CSS (Kitagawa, 2010; Sutton, 2014). Conflating these distinct pathologies leads to diagnostic errors and inappropriate therapeutic targeting.

### 10.2 Surgical risks: Carotid endarterectomy and stenting

The most acute clinical consequences of CB/CS conflation manifest during carotid interventions, where periprocedural hypotension/bradycardia from CS activation is a well‐recognized hazard (Reslan et al., 2022). Carotid endarterectomy (CEA) and carotid artery stenting (CAS) target atherosclerotic disease of the CB but carry the inherent risk of inadvertent CS stimulation (Qureshi et al., 1999; Tarlov et al., 1973). The central challenge is navigating the visible (CB geometry, plaque) while respecting the invisible (baroreceptor field distribution).

#### 10.2.1 Intraoperative hemodynamic instability

Surgical manipulation, dissection, clamping, and arterial stretch can trigger exaggerated baroreflex responses: profound bradycardia, asystole (>3 s), and severe hypotension compromising cerebral and coronary perfusion (Arhuidese et al., 2020; Bogniotti et al., 2022). Risk is amplified by variability in the baroreceptor field: a surgeon assuming standard ICA‐origin localization may be unprepared for severe reflexes when manipulating regions with greater baroreceptor concentration proximal or distal to the ICA (Bogniotti et al., 2022; Kikuta et al., 2019; Toorop et al., 2009). Many surgeons preemptively infiltrate local anesthetic (lidocaine) around the bifurcation to block the CSN, but effectiveness depends on accurately targeting the variable sensory field (Lee et al., 2024; Maher et al., 2002).

#### 10.2.2 Post‐procedural baroreflex dysfunction

Aggressive dissection or denervation can injure carotid baroreceptors or their afferent pathways and precipitate post‐operative baroreflex failure with severe, labile hypertension (Biaggioni et al., 2019; Timmers et al., 2003). Such hypertension is a recognized risk for bleeding complications, including suture‐line arterial bleeding and neck haematoma, after carotid surgery (Stoneham & Thompson, 2009). It also increases the risk of cerebral hyperperfusion syndrome (headache, seizures) and intracranial hemorrhage after CEA/CAS (Bouri et al., 2011; Lin & Liu, 2020; Opaskar et al., 2024).

#### 10.2.3 Stenting and baroreceptor field location

In CAS, balloon inflation and stent deployment stretch the arterial wall, eliciting baroreflex responses that depend on the stent's position relative to the baroreceptor field (Lavoie et al., 2008). Studies demonstrate that stenting the proximal CB “body” produces a significantly higher incidence of transient bradycardia/hypotension compared to distal “apical” stenting (Suh et al., 2012). This provides direct clinical evidence that the distribution of baroreceptors is non‐uniform and that neuroanatomical precision affects procedural outcomes.

### 10.3 Baroreflex activation therapy: Precision targeting of the carotid sinus

Baroreflex activation therapy (BAT) for resistant hypertension is a clear clinical example of why distinguishing the CB from the CS matters. Trials and reviews show that BAT lowers blood pressure in patients with resistant hypertension by electrically activating the carotid baroreflex, independent of plaque therapy at the CB (Bakris et al., 2012; Bisognano et al., 2011; Victor, 2015). BAT delivers electrical stimulation via an implanted carotid electrode to mimic elevated pressure signals, thereby reducing sympathetic tone and lowering arterial pressure (Alnima et al., 2015; Victor, 2015). Therapeutic success depends on precise electrode placement over the baroreceptor‐dense region, guided by intraoperative mapping rather than solely by the visible contour of the CB (Niu et al., 2019; Tordoir et al., 2007). Human immunohistochemistry shows the highest density of baroreceptors in the medial proximal ICA (Toorop et al., 2013). Regulatory and professional body guidance on implantation instructs surgeons to map the CS and position the lead at the site that elicits the strongest baroreflex response (Schmidli et al., 2007; Victor, 2015; Wilks et al., 2017). Accordingly, positioning based solely on CB geometry risks ineffective stimulation or suboptimal responses. In contrast, response‐guided mapping (testing multiple spots and fixing the lead at the site with the maximal systolic blood pressure/heart rate response) improves effectiveness (Kansal et al., 2016; Schmidli et al., 2007; Weaver et al., 2016). BAT thus exemplifies a paradigm shift from treating structural vascular disease (CB plaque/stenosis) to directly modulating a functional neuroregulatory circuit (the CS baroreflex), underscoring the clinical necessity of precise CS‐focused terminology as cardiovascular neuromodulation advances (Chatterjee & Singh, 2015).

### 10.4 Practical implications for reporting

To reduce ambiguity in imaging reports and operative documentation, atherosclerotic disease should be localized by vessel segment and wall orientation rather than by the nonspecific term carotid bulb (e.g., “eccentric plaque at the posterolateral origin of the ICA” or “plaque in the distal CCA extending to the proximal ICA”). The term CB should be used only as a geometric descriptor when its extent is explicitly stated (presence/absence and, where relevant, longitudinal length). In contrast, the CS should be reserved for baroreceptor‐related physiology (e.g., CSS, baroreflex responses, or autonomic neuromodulation) and should not be used to localize plaque.

## 11 LIMITATIONS OF THE CURRENT STUDY

This narrative review does not provide quantitative effect estimates. Interpretation of prior studies is limited by marked heterogeneity in how the CB and CS have been defined across anatomical, imaging, and clinical literature. Direct neurohistological mapping of CS baroreceptor distribution in humans remains sparse, necessitating partial reliance on animal data.

## 12 CONCLUSION AND A CALL FOR PRECISION

This review clarifies that the CB is a geometric dilation that shapes local hemodynamics and atherosclerosis risk. In contrast, the CS is a baroreceptor field that mediates the arterial baroreflex. Although they often co‐localize, CB size, presence, or position does not predict baroreceptor distribution or function. Clinically, two partly independent hazards should be recognized: embolic risk from posterolateral CB atherosclerosis and hemodynamic instability from medial CS dysfunction. We therefore recommend consistent terminology across clinical documents, imaging reports, and teaching materials: use CB for geometry/hemodynamics and CS for reflex physiology, stating their spatial relationship explicitly when both are relevant. Adopting this standard will reduce clinical ambiguity and align neurovascular research and care on a standard, functional map of the bifurcation.

## AUTHOR CONTRIBUTIONS

**Răzvan Costin Tudose:** Conceptualization; methodology; software; investigation; data curation; writing – original draft; writing – review and editing. **Nicolae Radu:** Software; investigation; data curation; visualization. **Mugurel Constantin Rusu:** Methodology; validation; resources; supervision; writing – review and editing.

## CONFLICT OF INTEREST STATEMENT

The authors declare no conflicts of interest.

## Data Availability

The datasets used and analyzed during the current study are available from the corresponding author upon reasonable request.
